# A Perspective on Roles Played by Immunosenescence in the Pathobiology of Alzheimer's Disease

**DOI:** 10.14336/AD.2020.0205

**Published:** 2020-12-01

**Authors:** Yan Zhao, Jun-Kun Zhan, Youshuo Liu

**Affiliations:** Department of Geriatrics, Institute of Aging and Geriatrics, the Second Xiangya Hospital, Central South University, Changsha, Hunan 410011, China

**Keywords:** immunosenescence, Alzheimer’s disease, inflammation, aging

## Abstract

Alzheimer's disease (AD) is a chronic progressive neurodegenerative disorder. Aging is the most significant risk factor for late-onset AD. The age-associated changes in the immune system are termed immunosenescence. A close connection between immunosenescence and AD is increasingly recognized. This article provides an overview of immunosenescence and evidence for its role in the pathogenesis of AD and possible mechanisms as well as the outlook for drug development.

## 1. Introduction

Alzheimer's disease (AD) is the most common type of dementia characterized by progressive memory loss, visual-spatial impairment, executive dysfunction, and personality and behavioral changes. According to the "Word Alzheimer Report 2018", there are nearly 50 million AD patients worldwide in 2018, and this number is expected to increase to 152 million by 2050. The pathological features of AD are neuritic plaques, neurofibrillary tangles, neuronal and synaptic loss, and the activation of microglia [1]. Over the past few decades, the amyloid cascade hypothesis has dominated the field of AD research, suggesting that Aβ deposition is the central event in AD pathology [2]. However, recent findings have challenged this hypothesis and argue that Aβ protects the brain from infection, and its aggregation promotes microglia-mediated neuroinflammation [3]. The viewpoint that altered immune and inflammatory responses may play the main role in the progression of AD has increasingly been recognized [4, 5].

Aging is characterized by a time-dependent loss of anatomic and physiological integrity,resulting in increased vulnerability to some diseases and death [6]. Induced by genetic, epigenetic, and environmental factors [7], aging affects almost all organs and has a profound impact on the immune system [8]. AD is the most common type of age-related neuronal disorder[9]. It is hypothesized that AD pathology is regulated by the immune system in an age-dependent manner [10].

In recent years, research is making significant progress and proposes that immunosenescence actively participates in the pathogenesis of AD and mediates inflammatory damage. However, it is still unclear how specific changes in the immune system with aging affect the central nervous system (CNS) and cause AD. In this review, we provide an overview of changes in the immune system with aging, but this is not a comprehensive review of immunosenescence. We mainly discuss the changes related to AD and improvement strategies.

## 2. Immunosenescence

The immune system is responsible for preventing the invasion of pathogens and removing damaged or harmful cells, like senescent cells and toxic substances. Immunosenescence includes degeneration of immune organs, aging-related changes in the phenotype and function of immune cell subsets, and chronic inflammatory states. Although immunosenescence occurs in almost all aged people, differences in genetics, environment, lifestyle, and nutrition cause heterogeneity among different individuals, making some people more susceptible to developing certain diseases [11]. For example, immunity is relatively stable in some populations, retaining more young immunological parameters. Offspring of Sicilian centenarians found that they retained more young immune parameters, suggesting that genetic or environmental factors can delay immunosenescence[11]. A growing body of studies has provided supportive evidence for the role of immunosenescence in contributing to AD [12, 13].

## 3. Roles played by immunosenescence of innate immune in the pathology of AD

### 3.1 Immune Barrier

The immune barrier is a physiological and anatomical structure that prevents pathogens or molecules from entering the body or parts of the body. It is an important component of non-specific immune function and is the first line of defense. The immune barriers include skin, respiratory mucosa, digestive tract mucosa, placental barrier and blood-brain barrier (BBB), etc. The immune barrier plays a protective role through a physical barrier, chemical killing and bio-antagonism. Aging leads to barrier dysfunction and increases the challenge of the innate immune system in the elderly. Living together with diverse microbes, there are trillions of microbes in our tissues such as intestines and lungs and other tissues. Recent studies have revealed the dynamic interaction between the microbiota and the immune system, as well as the pathogenic results of maladjustment [14].

#### 3.1.1 Gut barrier

The gut barrier is a structure that separates the environment and the host’s interior, and regulates the selective passage of substances, as well as promotes the absorption of nutrients, while prevents the invasion of pathogens and other toxic substances from entering the human body [15]. Under homeostatic conditions, gut microorganisms regulate mucus secretion and enhance the integrity of the intestinal barrier by producing short-chain fatty acids [16]. Lymphocytes such as Th17 participate in host defense responses by producing IL-22. Epithelial cells produce antimicrobial peptides under the stimulation of interleukins [16]. Dendritic cells induce the activation and differentiation of naive B cells to produce plasma cells that secrete specific IgA in the lamina propria [17]. IgA is transported into the gut lumen in the form of sIgA, which binds to commensal microorganisms and soluble antigens, inhibiting their adhesion to epithelial cell surfaces and intestinal barrier penetration [18].

An important feature of the alimentary tract is immune tolerance, allowing certain bacteria to reside in specific areas [19]. However, if any of these resident bacteria migrate to other places, it may present a potential hazard [20]. In addition to actively selecting a more suitable environment, bacteria may also accumulate due to immunosenescence [20]. Studies have attempted to explore the connection between aging and the gut barrier, which found that permeability of the intestinal barrier changed with age. Changes in contractile force and innervation of the intestinal smooth muscle of elderly rodents [21], as well as increased permeability of the intestinal tract to macromolecules [22], indicate that aging is associated with deterioration in the function and integrity of the intestinal barrier. In the aging population, the altered microbiota composition leads to increased adhesion and leakage of microorganisms and microbial by-products through the gut barrier,eventually causing the occurrence of disease [23]. The gut microflora of the elderly is different from that of the healthy adults, which is partly due to aging, such as weakened immunity, physiological and morphological changes in the gut, altered lifestyle and dietary structure, *etc* [23, 24]. Age-related changes in the microbial-intestinal-brain axis lead to increased circulating inflammatory factors, which can also be seen in mice [25].

Researchers have found that intestinal flora is closely linked to diseases such as metabolic diseases, autism, depression, Parkinson's disease(PD), and AD [26]. In 2016, scientists confirmed for the first time the link between the intestinal flora and PD and found that the production of short-chain fatty acids by the intestinal flora can activate brain microglia, which causes neuroinflammation and neuronal damage [27]. This year, researchers at Johns Hopkins University in the United States confirmed that misfolded α-synuclein can travel from the small intestine to the brain along the vagus nerve, and it can prevent α-synuclein from entering the brain by cutting off the vagus nerve, which may become the key to prevent PD [28].

To explore the correlation between microbial flora and AD, the intestinal microbiota of AD patients and age- and sex-matched control individuals was investigated. The AD group has reduced microbial diversity and has a unique bacterial genus,which related to changes in cerebrospinal fluid (CSF) biomarkers [29]. Neuro- pathological studies found that the levels of LPS and *E. coli* K99 pili protein were higher in AD brain parenchyma compared to controls[30]. What’s more, LPS colocalized with Aβ in amyloid plaques and vessel walls in AD brains, suggesting that the displacement of the intestinal flora may occur in AD patients [30].

How do intestinal pathogens enter the brain tissue? One possibility is that the gut microbes and highly pro-inflammatory neurotoxins reach the circulation from the gut mucosa by a special adhesion structure,and then enter tissues and organs,including the brain tissue[31]. Microorganisms may also reach the brain tissue through the vagus nerve, causing a series of pathological changes. Among these mechanisms, direct regulation of gut microbiota may reduce the inflammatory response and improve immune response,and finally prevent disease [32].

#### 3.1.2 Oral and nasal mucosa

A large number of bacteria are found in the oral and nasal mucosa, and various types of bacteria form a complex ecosystem [33]. There is a dynamic equilibrium between bacteria in dental plaque and host immunity [34]. Bacterial load is likely to increase with age [35]. Increasing evidence suggests that there is a link between AD and periodontitis. On the one hand, AD patients are more susceptible to periodontitis. On the other hand, patients with periodontitis are more likely to suffer from AD than those with a healthy periodontal condition, and the severity of periodontitis is positively correlated with the decline in cognitive impairment [36]. A meta-analysis in 2017 showed that the risk of AD in patients with periodontitis was almost three times that of non-periodontitis patients, and the oral health performance of AD patients was also worse than that of non-AD patients [37]. Periodontitis may affect the brain through intact pathogenic microorganisms or inflammatory mediators. Some nerves originate from brain tissue and distribute in the oral or nasal mucosa, including the trigeminal and olfactory nerves. The pathogens or other harmful molecules may invade the CNS along the olfactory pathway or the trigeminal nerve, or enter the systemic circulation through the mucous membrane and finally reach the CNS [38]. It has been confirmed that there is oral Treponema in the trigeminal nerve and may be the pathway for oral pathogens in AD to enter the brain [39]. Olfactory ensheathing cells (OECs) provide bactericidal protection against invasion of pathogenic microorganisms from the nose and mouth [39]. They have macrophage-like functions such as expressing inducible carbon monoxide synthase when attacked, and then engulf bacteria and migrate [39]. OECs have been proven to bypass the BBB and successfully transfer drugs containing nanoparticles to the brain [40]. The above evidence suggests that it may act as a channel for pathogens or other environmental stimuli to enter, and then spread through the olfactory pathway to pathological changes throughout the brain.

#### 3.1.3 BBB

BBB is a highly selective barrier between blood and brain tissue, which restricts the uncontrolled diffusion of cells and molecules from the blood into CNS, and regulates nutrients into brain tissue [41]. In the context of aging or disease, potentially harmful proteins may penetrate the brain through the damaged BBB [42]. Recent evidence shows that BBB breakdown occurs in healthy aging, and is worse in individuals with mild cognitive impairment [42]. BBB disruption is considered a biomarker of early cognitive damage, which is independent of Aβ and tau pathology [43].

Tight junction proteins connect the endothelial cells of brain capillaries,preventing molecules in the plasma from entering the brain [44]. Reduced expression of tight junctions (TJs) in aged mice inhibits the communication between endothelial cells and increases the permeability of BBB [45]. Overactivation of the peripheral immune system, determines the increased release of pro-inflammatory cytokines and chemokines, and up-regulate the expression of immune receptors. These immune molecules, reach the CNS through the damaged BBB, causing brain tissue damage [46]. During AD pathology, damaged brain and high permeability of BBB determine that peripheral immune cells may participate in CNS immune response, as autopsy showed that T cells infiltrated into brain tissue [47]. Besides, monocytes also enter the brain through BBB to form macrophages or dendritic cells, with different states of activation[48]. Integrity destruction of BBB in AD patients partly reveals the clues of migration of peripheral immune cells to the damaged brain [49].

There are transport systems on the BBB that mediate the transfer of Aβ into and out of the brain. P-gp and LRP-1 are important efflux transporters that mediate the clearance of Aβ from brain tissue [50]. P-gp function decreased in both aged humans [51, 52]and in aged mice [53]. The expression level of LRP1 is reduced in cerebrovascular endothelial cells in normal aging and AD patients [54]. Further study showed that the expression level of LRP1 in microvasculature was negatively correlated with Aβ accumulation [54]. Collectively, these changes of transporters on BBB with age may lead to the accumulation of Aβ in the AD brain.

Glucose is the most important energy source for brain tissue. Glucose transporter 1 (GLUT1) on BBB vascular endothelial cells,a unidirectional transporter, is a receptor for glucose transport [55]. Studies have shown that the reduction in glucose uptake by the brain could reflect the dysfunction of BBB [56]. GLUT1 reduction at BBB was found in 15-month-old mice, and reduce more in AD models of the same age [57]. PET-CT can measure the brain glucose uptake, and there is reduced glucose uptake in the frontal and temporal lobe of the elderly [58]. Aged rodents also show a reduction of brain glucose uptake, which is related to cognitive impairment [59].

Increased BBB permeability with age makes it possible for microorganisms and their products to enter the CNS. At the same time, changes in multiple transporters expressed on aged BBB lead to malabsorption of nutrients and reduced Aβ efflux, eventually contribute to the pathology of AD.

#### 3.1.4 Choroid plexus

Choroid plexus (CP), as the site of the blood-CSF barrier, is recognized to provide a protective effort for the brain. Due to its anatomical structure and physiological characteristics, CP restricts the migration of immune cells and plays an important role in maintaining brain homeostasis. In the CP matrix, researchers have found almost all kinds of immune cells, and the traffic of these cells depends on signals generated by the CNS, such as TNFa and IFNγ [60]. In aging CP, an increased Th1:Th2 ratio leads to increased expression of the chemokine CCL11 and decreased permeability to leukocytes [61]. Besides, CCL11 is thought to promote the transformation of microglia into an inflammatory state [62], which is associated with cognitive impairment. Furthermore,it was found that the anti-inflammatory factor IL-4 was elevated in the aging CP, which induces the CP epithelial cells to produce more CCL11 [62]. Another study by the same team showed that an increase of IFN1 in CP in aged mice offsets the effects of CCL11 and reduces age-related chronic inflammatory processes [63]. Therefore, we speculate that aging affects the balance of peripheral immune cells that homing to the brain and thus participates in the regulation of CNS function.

During disease progression,researchers found that IFNγ levels at the CP decreased in 5×FAD mice, APP/PS1 mice and brain aging [64], as well as other neurodegenerative diseases [65]. IFN γ is mainly produced by Th1 cells, and Tregs can inhibit Th1 cell function [64]. By transient Treg depletion, the IFN γ signal pathway in CP is restored, which increases peripheral immune cells transfer to the brain parenchyma that may clear Aβ, and finally improve memory and behavioral disorders [66]. The above results show that the selective trafficking of immune cells via the CP is one of the underlying mechanisms of pathological changes in neurodegenerative diseases [67].

### 3.2 Immunosenescence of innate immune cells Microglia

Microglia are innate immune cells that reside in the brain and play an important role in maintaining homeostasis and immune defense [68]. When there are no harmful stimuli, the microglia remain stationary, with poor replication ability. Although in a relatively static state, they still extend and retract their protuberances to screen and respond to dangerous challenges [69].

Microglia undergo significant changes in the aging brain. Morphologically, aged microglia exhibit cytoplasmic hypertrophy and branch reduction [70]. Functionally, senescent microglia show higher proliferative capacity and production of proinflammatory cytokines, but reduced chemotaxis and ability to clear Aβ [71]. After peripheral injection of lipopolysaccharide, aged mice showed increased expression of pro-inflammatory factors and increased activity of microglia [72]. In addition, in vitro experiments revealed that senescent microglia secrete more TNF-α and IL-6 than that of young mice [73].

Activated and proliferated microglia surround amyloid plaques in the AD brain and participate in the clearance of Aβ [74]. Microglia clear the dissolved Aβ via a pinocytosis form or an LDL receptor-associated protein-mediated pathway [75]. Insoluble Aβ binds to the receptors on the surface of microglial cell membrane. Aβ binds to TLRs, RAGE and other receptors on the surface of microglia membranes, transducing intracellular signaling pathways, then leading to the synthesis and release of pro-inflammatory factors [75]. In the aging brain, the phagocytic capacity of microglia is weakened, which leads to the accumulation of Aβ. Microglia continue to activate, leading to chronic inflammation, increased oxygen free radicals, mitochondrial damage, and ultimately neuronal death [76].

#### Monocyte/Macrophage

Monocytes can differentiate into macrophages, dendritic cells, and antigen-presenting cells. Infiltrating monocytes express pattern recognition receptors that detect substances released by damaged CNS cells or recognize surface antigens of invade pathogens [69]. Upon recognition of these molecules, a pro-inflammatory signaling cascade can be induced, and infiltrating monocytes acquire an activated phenotype, exhibiting a cytoplasmic enlarged amoeba morphology [77]. During aging, both microglia and macrophages have defects in phagocytosis and chemotaxis [78]. As to pro-inflammatory response, senescent macrophages appear unlikely to produce functional pro-inflammatory responses [69].

The exact source of amoebic myeloid cells surrounding amyloid plaques has been controversial due to the technical challenge of identifying invasive myeloid cells and locally activated microglia. Recent evidence suggests that peripherally derived macrophages maintain unique functional and transcriptional features in the CNS [79]. After entering the AD brain, monocytes become the most important cells that phagocytose Aβ [80]. Monocyte-derived macrophages are very effective in binding to Aβ, as confocal microscopy revealed that the MHC-II/Aβ42 complexes were only present on AD monocytes/macrophages [81]. Activated macrophages exhibit two phenotypes, namely M1 and M2, depending on the local microenvironment [82]. M1 produces pro-inflammatory factors and M2 is associated with the production of regulatory or anti-inflammatory factors [69]. M1 and M2 are balanced in healthy people, but in the presence of inflamm-aging, there is an imbalance,contributing to age-related disease development [83]. Researchers proposed macrophages as key cells in the induction and maintenance of inflamm-aging [84]. Under the induction of Aβ, macrophages change their phenotype M2 to phenotype M1, which produces IL-1b and TNFa. Macrophages interact with microglia and amyloid plaques and are thought to be inflammatory activators that produce cytokines and reactive oxygen species (ROS), leading to neuronal loss and apoptosis [34].

#### Natural killer cells

NK cells, different from T cells and B cells,are defined as innate cytotoxic lymphocytes [85]. According to the expression of phenotypes and functional markers, NK cells are divided into two types: CD56^bright^ and CD56^dim^[86]. CD56^bright^ has an immunomodulatory role and can produce high levels of cytokines and chemokines [87]. 3xTgAD mice developed premature immunosenescence at 4 months of age, while NK percentage and cytotoxic activity changed at the age of 2 months, well before amyloid plaques and cognitive impairment [88]. There is no difference in the distribution of NK cells in the age-matched healthy elderly patients with AD, but the phenotype and function of NK cells have changed in the amnestic MCI (early stage of AD), showing the activation state and excessive cytokine secretion [89]. Changes of NK cells may be one of the peripheral markers in the preclinical or prodromal phase of AD [90].

## 4. Roles played by immunosenescence of adaptive immune in the pathology of AD

In contrast to the activation of innate immunity,adaptive immunity appears to be much less prominent in AD pathology [91]. The main cellular components of adaptive immunity are T and B lymphocytes. The adaptive immune response plays a key role in the development of control against pathogens and toxic molecules including misfolded tau and Aβ proteins [92].

### 4.1 T cells

T cells are mainly involved in cellular immune response, which is important for anti-tumor, transplant rejection and delayed-type hypersensitivity. T cells and their secreted cytokines are important for protecting brain function homeostasis. A series of studies have established the ability of CD4^+^ T cells to maintain cognition and behavior in naive mice, especially CD4^+^ Th cells and their secreted cytokines, which promote memory function and social behavior [34]. Among them, IFNγ produced by CD4^+^ Th1 cells residing in the meninges supports neural circuits and is very important for social behavior [93]. IL-4 produced by CD4^+^ Th2 regulates meningeal dendritic cells and stimulates astrocytes to produce BDNF, which promotes learning ability [94].

Senescent T cells have fewer T cell receptors and may turn into dysfunctional "virtual memory" cell phenotypes [95]. In vitro studies indicated that CD4^+^ naive T cells derived from the elderly and aged mice have decreased proliferative activity, altered ability to secrete cytokines, and reduced reactivity to TCR stimulation [96]. Similarly, aging will affect the ability of CD8^+^ T cells to aggregate. Another important feature of immunosenescence of T cells is the increase in highly differentiated memory CD8^+^ cells stimulated by chronic viruses such as CMV [96]. Terminally differentiated senescent T cell expansion, and over expression of cytokines and pro-inflammatory factors, will result in a host with high inflammatory response [34]. Compared with middle-aged control individuals, Aβ-reactive T cells were easily detectable in the peripheral blood of AD patients and healthy elderly subjects [97]. Spleen is the biggest peripheral immune organ. It was noted that there was a higher frequency of Tregs in splenocytes derived from old transgenic mice [98]. Transient depletion of Foxp3^+^ Tregs or pharmacological inhibition of Treg activity was associated with reduced neuroinflammatory response, amyloid plaque clearance and cognitive improvement [99]. Treg depletion was also shown to influence the choroid plexus and as such affect CNS traffic of immune cell that included Tregs and monocyte-derived macrophages to plaque sites [100-101].

### 4.2 B cells

B cells are the only cells that produce antibodies, and they are also full-time antigen-presenting cells. Immuno-senescence of B cell is characterized by less receptor diversity and weakened ability to convert to memory B cells, along with reduced antibodies response to antigens [102]. The decrease of antibody specificity and affinity affected by age will increase the susceptibility of diseases in the elderly [103]. What’s more, the ability of B cells to convert IgM to IgG, IgE or IgA is reduced during aging, both in humans and mouse models [104]. A specific set of mature B-lymphocyte subsets related to age may aggregate in elderly individuals, which may promote inflammation and autoimmune response [34].

**Table 1:** Alterations in the cellular components of innate and adaptive immunity associated with aging.

Immune cells or their products	Changes related to AD pathology	Ref.
Innate immunity		
Microglia	Ability to phagocytose Aβ fibrils ↓	[71]
	Production of proinflammatory cytokines ↑	[72,73]
Monocyte/Macrophage	Functional proinflammatory response ↓	[69]
	Phagocytosis and chemotaxis ↓	[78]
	The imbalance between M1 and M2 ↑	[83]
Natural killer cells	Number of NK cells and cytotoxic activity ↓	[88]
Adaptive immunity		
T cells		
Th1/IFNγ	IFNγ signaling activation supports neural circuits ↓	[93]
Th2/IL-4	IL-4 stimulates astrocytes to produce BDNF ↓	[94]
Treg cells	Frequency of Treg cells ↑	[98]
B cells	Antibody specificity and affinity ↓	[103]
	Aβ antibody levels ↓	[108]

The research on whether B cells participate in the pathogenesis of AD is limited. Reductions in the number of peripheral B cell subsets were found in some AD patients [105]. Misfolded Aβ peptides have been shown to induce B cell-mediated immune responses,generating autoantibodies against Aβ being found in the CSF and blood [106]. These autoantibodies seem to share conformation-specific binding epitopes and promote microglia-mediated clearance of amyloid plaques [107]. There are antibodies against different forms of Aβ in healthy individuals, and these antibody levels decrease with normal aging. In addition,with aggravation of AD, the downward trend is more pronounced [108]. In the acute phase of CAA-RI, anti-Aβ antibodies are elevated, and after clinical remission, autoantibodies gradually decrease,suggesting that the effect of B cells on amyloid pathology is complex [109].

In summary, age-related changes in innate and adaptive immune cells and their secreted cytokines are involved in the pathogenesis of AD (Table 1).

## 5. Inflamm-aging in AD

Inflammation, a normal repair response, is crucial to combat pathogens and clear dead cells. The initiation and dissipation of physiological inflammatory responses are strictly regulated [110]. Once the inflammation is dysregulated, it will cause tissue damage. Inflamm-aging refers to a state of chronic pro-inflammatory response in the process of aging,which is controllable, asymptomatic, chronic and systemic, and is considered to be a part of immunosenescence [111]. The basis of inflamm-aging is the remodeling of the immune system, characterized by increased levels of cytokines and other pro-inflammatory markers in the circulation [112], such as a 2-4 fold increase in serum IL-6 and CRP [99, 100][113].

AD is also considered to be a chronic inflammatory disease. The inflammatory response of AD is not limited in the brain, but also exists in peripheral tissues, which is considered to be part of the systemic inflammatory response [60]. In AD mice, it was found that elevated levels of pro-inflammatory factors in the circulation are significantly associated with cognitive decline [114]. A prospective study of 1633 participants showed a positive correlation between peripheral inflammatory response in middle age and late brain volume reduction [115]. AD often coexists with chronic inflammatory diseases such as diabetes mellitus and rheumatoid arthritis (RA) and psoriasis [116]. TNFa is a key cytokine that drives the pathogenesis of RA, and RA patients reduce the risk of AD after receiving anti-inflammatory treatment [117]. At the same time, peripheral neutralization of TNFa is considered to be effective in animal models of AD.

The chronic inflammatory state in aging individuals may be associated with long-term chronic microbial infections,which may be a driver of cognitive decline and possibly dementia in the elderly [118]. Candidate pathogens proposed over the years include herpes simplex virus type 1(HSV-1), herpes simplex virus type 2 (HSV-2), human herpesvirus 4 (HHV-4), human herpesvirus 5 (HHV-5), human herpesvirus 6 (HHV-6) and 7 (HHV-7), intestinal bacteria (*H.pylori*), periodontal bacteria (*P.gingivalis*), spirochetes (*B.burgdorferi*) and other bacteria [119]. These pathogens may invade CNS via the trigeminal nerve, olfactory nerve, gastrointestinal tract, and BBB. They also cause an inflammatory reaction in the periphery, which circulates to the brain through the blood, causing central inflammation [38].

**Figure 1.**
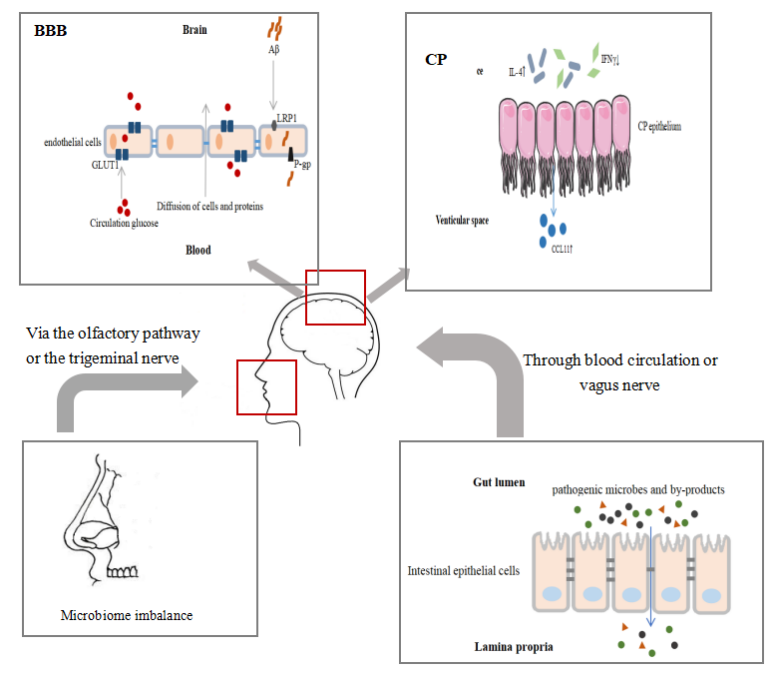
Schematic representation of age-related changes of immune barriers. Studies have shown that changes in immune barriers such as permeability and receptor expression, increasing challenges of the innate immune system, which are associated with the pathology of AD. Selective trafficking of immune cells via the immune barrier is also one of the underlying mechanisms of pathological changes in neurodegenerative diseases.

## 6. Novel treatments for AD

There is no disease-modifying drug available for AD, and current symptomatic treatment provides limited help. As described above,we put forward the possibility that immunosenescence is a contributing cause of AD. Reversing immunosenescence to restore immune protection in the CNS is a promising strategy for AD therapy (Fig. 1).

### 6.1 Antibacterial and antiviral treatment

**Figure 2.**
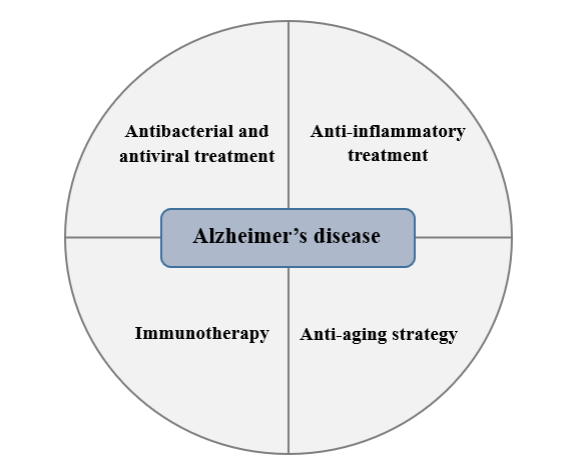
A close connection between immunosenescence and AD is increasingly recognized. New treatments for AD aiming at regulating immunosenescence may relieve neurodegeneration. As to the imbalance of barrier flora, chronic low-grade inflammation and aging, antibacterial, antiviral and anti-inflammatory treatment, immunotherapy, and anti-aging strategy may become new methods to treat AD.

Alzheimer's disease has been thought to be associated with viral or bacterial infections for a long time and is supported by increasing evidence [120]. Pathogen invasion into the brain may act as a trigger or aggravating factor for AD. As mentioned above, pathogens in the mouth, nose or intestines may enter the CNS through the damaged intestinal barrier, oral and nasal mucosa, or neural access (Fig. 2). In addition, chronic microbial infection releases pro-inflammatory substances, which reach vascular circulation and cross BBB to cause neuroinflammation [34]. In AD mouse and worm models, Aβ can aggregate into a network to prevent Salmonella or Candida infection [121]. Long-term broad-spectrum antibiotic treatment reduces amyloid plaque in APP/PS1 mice and attenuates the local glial reactivity around plaques, as well as alters the morphology of microglia [122]. Numerous studies have demonstrated the key role of gut dysbiosis in neurodegeneration. Disturbances along the brain-gut-microbiota axis may be involved in the pathogenesis of AD [123]. GV-971, a new drug approved in 2019 in China to treat AD, can regulate gut flora imbalance and reshape immune homeostasis. It can also prevent the infiltration of peripheral immune cells into the brain, inhibit neuroinflammation and prevent the progression of AD [124]. Treatment with antivirals and/or antibacterials may alleviate AD pathology. Studies found that antiviral therapy in subjects with HHV-5 or HSV could be beneficial in reducing the incidence of AD [125, 126]. However, there is no epidemiological data suggest the hypothesis that antibiotic use decreases the risk of AD onset. But if we know which bacterial plural are overexpressed in the mouth, nasal cavity and gut of AD patient, suitable antimicrobial agents may benefit them [127].

### 6.2 Anti-inflammatory treatment

Consistent with the concept that inflamm-aging may induce or aggravate the pathogenesis of AD, long-term administration of anti-inflammatory drugs may reduce the risk of AD [128]. However, clinical trials of non-steroidal anti-inflammatory drugs (NSAIDs), such as Naproxen, Celecoxib and Rofecoxib showed no clinical benefit in the treatment of AD [129]. There are conflicting opinions about the use of NSAIDs in AD. Further Randomized Controlled Trials (RCTs) should be conducted with larger samples for a long duration to study the role of NSAIDs or other anti-inflammatory drugs in the treatment of AD [130].

### 6.3 Immunotherapy

Immunotherapies against Aβ and tau are currently the main tested therapeutic approaches. According to the latest statistics in 2019, among the 132 agents in AD clinical trials, 40% of the drugs are aimed at ameliorating the amyloid pathology, of which 20 are monoclonal or biological therapies [131]. Seventeen percent have tau as the target,and there are ten biologics among anti-tau agents [131]. Moreover, a plethora of immunomodulators such as cytokines, complement components, and histocompatibility proteins are essential for functional synaptic formation [132]. However, it is still unclear whether boosting or suppressing the immune system, in the brain or the periphery, may reverse neurodegeneration in AD patients [133]. Whether immunotherapy can stimulate the infiltration of immune cells and factors into the brain to regulate inflammation needs further research to confirm.

### 6.4 Anti-aging strategy

In addition to a specific treatment, slowing down age-related systemic decline will benefit AD. Researchers proposed that geroprotectors,compounds that slow the rate of biological aging, may reduce the incidence of AD, which are likely to be novel AD drug candidates [134]. Animal experiments have confirmed that young blood can change the cognitive state of old mice [135]. A Phase I clinical trial using younger blood to improve mild to moderate cognitive impairment is considered safe and tolerable, and its effect is worth looking forward to [136]. As an anti-aging strategy,inhibition of mTOR ameliorates immunosenescence in old humans, and also suppresses brain aging to prevent neurodegeneration [137].

Table 2 lists the new drugs and their specific mechanisms for treating AD. The task to develop an effective treatment for AD is arduous, but strategies harnessing our knowledge of immunosenescence may become new methods to treat AD.

## 7. Conclusion

Over the decades, there is enormous progress in describing the immune age-related alterations. With age, the inherent immune and adaptive immune defects increase the vulnerability of the immune system, which may lead to cognitive impairment [127]. Immuno-senescence has emerged as a crucial player in the pathogenesis of AD, but its functional role remains unclear. The new era forces us to explore how the neuro-immune system communicates with each other in the context of aging, and further decipher the effect of immunosenescence on the brain. Aiming at regulating the changes of the age-related immune barrier, eliminating aging immune cells, and fighting chronic inflammation, we will expand the research perspective of AD.

**Table 2:** Novel treatments for AD to reverse immunosenescence.

Drug classification	agent	Mechanism of action	Ref.
Antimicrobial therapy	Antibiotic treatment	Avoid bacterial infections	[122]
	Antiviral agent	Protects against virus infections	[125,126]
	GV-971	Regulate gut flora imbalance and reshape immune homeostasis	[124]
Anti-inflammatory treatment	Anti-inflammatory drugs	Modulate inflammatory processes	[128]
Immunotherapy	Antiamyloid agents	Remove amyloid and prevent amyloid production and aggregation	[131]
	Anti-tau agents	Reduce tau-mediated neuronal damage	[131]
immunomodulators	cytokines, complement components, and histocompatibility proteins	Improve cell signaling	[132]
Anti-aging strategy	Geroprotectors	Slow the rate of biological aging	[134]
	Young blood	Modulate aging and rejuvenate organs	[135,136]
	Inhibition of mTOR	Improve vaccine response	[137]
